# A multicentre review comparing long term outcomes of endoscopic vein harvesting versus open vein harvesting for coronary artery bypass surgery [version 1; peer review: 1 approved, 2 approved with reservations]

**DOI:** 10.3310/nihropenres.13215.1

**Published:** 2021-07-08

**Authors:** Bhuvaneswari Krishnamoorthy, Joesph Zacharias, William R. Critchley, Melissa Rochon, Iryna Stalpinskaya, Azita Rajai, Rajamiyer V. Venkateswaran, Shahzad G. Raja, Toufan Bahrami

**Affiliations:** 1 Department of Allied Health Professions, Faculty of Health and Social Service, Edgehill University, Ormskirk, UK; 2 Department of Cardiovascular Sciences, Faculty of Health, Biology and Medicine, The University of Manchester, Manchester, UK; 3 Department of Cardiothoracic surgery, Manchester Foundation Trust, Wythenshawe Hospital, Manchester, UK; 4 Department of Cardiothoracic surgery, Blackpool Victoria NHS Trust, Blackpool, UK; 5 Endothelial Cell Biology, School of Molecular Medicine, University of Leeds, Leeds, England, UK; 6 Department of Cardiothoracic surgery, Royal Brompton & Harefield NHS Trust, London, UK; 7 Centre for Biostatitics, Division of Population Health, The University of Manchester, Manchester, UK

**Keywords:** Open vein harvesting, survival, yendoscopic vein harvesting, major adverse cardiac events, coronary artery bypass graft, clinical outcomes

## Abstract

**Background:**

Utilisation of the Endoscopic Vein Harvesting (EVH) technique has been increasing for coronary artery bypass grafting (CABG) for the last two decades. Some surgeons remain concerned about the long-term patency of the long saphenous vein harvested endoscopically compared to traditional Open Vein Harvesting (OVH). The aim of this study was to perform a retrospective analysis of the outcomes between EVH and OVH from three UK centres with 10 years follow-up.

**Methods:**

27,024 patients underwent CABG with long saphenous vein harvested by EVH (n=13,794) or OVH (n=13,230) in three UK centres between 2007 and 2019. Propensity modelling was used to calculate the Inverse Probability of Treatment Weights (IPTW). The primary endpoint was mortality from all causes and secondary endpoints were length of hospital stay, postoperative complications, and incidence of repeat coronary re-vascularisation for symptomatic patients. IPTW was used to balance the two intervention groups for baseline and preoperative co-morbidities.

**Results:**

Median follow-up time was 4.54 years for EVH and 6.00 years for OVH. Death from any cause occurred in 13.8% of the EVH group versus 20.8% in the OVH group over the follow-up period. The hazard ratio of death (EVH to OVH) was 0.823 (95% CI: 0.767, 0.884). Length of hospital stay was similar between the groups (p=0.86). Post-operative pulmonary complications were more common in EVH vs OVH (14.7% vs. 12.8%, p<0.001), but repeat coronary re-vascularisation was similar between the groups.

**Conclusion:**

This large retrospective multicentre analysis indicates that EVH has a lower risk of mortality compared with OVH during the follow-up period of the study. The observed benefits of EVH may outweigh the risks but should be considered on a case-by-case basis. We hope this review gives confidence to other cardiac centres that offering an EVH approach to conduit harvesting does not affect long term patient outcomes.

## Introduction

Coronary artery bypass grafting (CABG) surgery with the long saphenous vein conduit is widely used to treat multiple coronary artery disease^1^. The long saphenous vein grafts have a reported higher incidence of early and late failure compared to arterial grafts due their different structural and functional properties^2–4^. One of the main factors contributing to early vein graft failure is damage to the endothelium, impact on the vasa vasorum and vascular nerves in the adventitia during harvesting^5^. Endoscopic vein harvesting performed by experienced practitioners provide good quality grafts compared to those obtained by those still learning the technique^3,6,7^. Current literature supports that handling during harvesting has an impact on the quality of the vein grafts if it is harvested by an inexperienced practitioner^2,8^. However, there are other factors which also have strong influence on the success of these vein grafts, including target vessels, progressive native artery atherosclerosis, patient co-morbidities, experience of the surgeon grafting and patient lifestyle^9,10^. Currently, there are no studies with long term follow-up of patients with comparison of outcomes between EVH and OVH in the United Kingdom (UK). We have analysed outcomes data from surgery performed at three cardiac centres in the UK over a 12-year period. Our primary endpoint was mortality from all causes and secondary endpoints were postoperative complications, length of hospital stays, and reinterventions for symptomatic patients.

## Methods

### Patients

**Figure 1:**
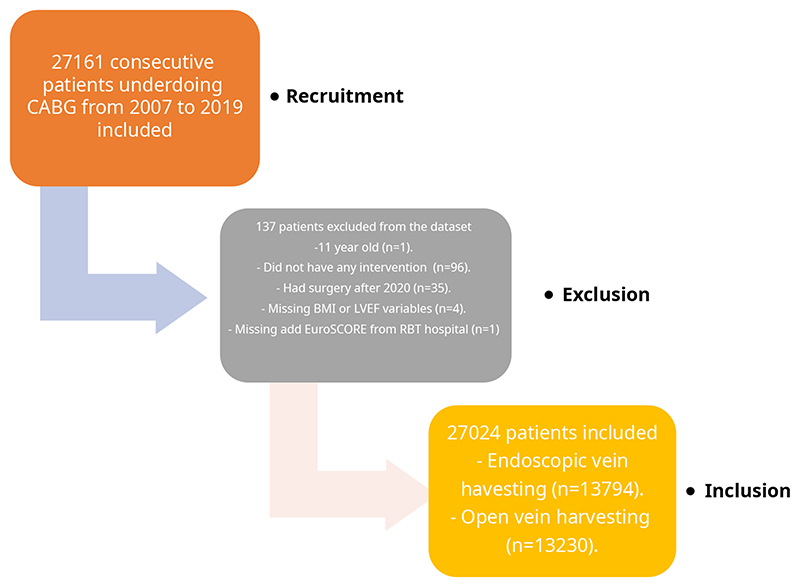
Patient recruitment and flow chart.

Between January 2007 and December 2019, 27,024 patients (OVH, n=13,230; EVH, n=13,794) who had CABG surgery with at least one long saphenous vein from Royal Brompton and Harefield (n=14,717), Wythenshawe (n=6,360) and Blackpool (n=5,947) hospitals were included in this study (Figure 1). All patients’ demographics, intraoperative and postoperative details were prospectively collected and entered into the database by healthcare practitioners. Data was obtained from a prospectively maintained institutional registry (Dendrite Clinical Systems, Henley-on Thames, UK) and NICOR UK database. In addition to this database, some of the intervention details were obtained from local cardiology centres and General Practitioner letters. All mortality and survival data were obtained from the national censor database, UK.

### Ethical approval

The Edge Hill University Health Research ethical committee (University REC ref: ETH2021-0066) approved this audit study protocol, which is in accordance with the principles of the Declaration of Helsinki. The ethical committee waived the need to obtain informed consent from the patients. In addition, the use of these data for this study was approved by the Manchester Foundation NHS Trust hospital institutional review board (registration no: 9477) and it was registered in the clinical governance audit departments. Only symptomatic patients who came back to hospital for reintervention were analysed as a secondary analysis for this study. All three centres cover large geographical areas and patients who get treated outside of the territory were unable to be included in this analysis.

### Surgical techniques

Data was included from patients whose vein was retrieved by experienced practitioners (previous experience of a minimum of 100 OVH cases and 50 EVH cases) at three hospitals. However, the Blackpool EVH dataset included patients’ outcomes following surgery by harvesters transitioning from OVH to EVH during the period of this study. The surgical techniques for open vein and endoscopic vein harvesting are standard surgical practice, as explained in previous publications^3,11^. All EVH patients received at least 2500 to 5000iu heparin before the start of EVH to avoid any intra-luminal clot formation^11^. Harefield and Wythenshawe hospitals used the Maquet Vasoview® Hemopro 1 and 2 (MAQUET, Inc, Wayne, NJ) vein harvesting systems. Blackpool hospitals have also used Maquet® Vasoview Hemopro 1 and 2 for 60% of their retrievals, and the remaining 40% were using the Terumo® VirtuoSaph (Terumo Cardiovascular, Ann Arbor, Mich).

### Study end point

The primary end points of this study were mortality, and the secondary end points were postoperative complications, length of hospital stay and repeat revascularisation for symptomatic patients.

### Statistical analyses

All patient characteristics were described by using count (percentage) for categorial variables and mean (standard deviation) for continuous variables. The primary outcome measure was death from any cause after surgery. Patients were followed from the date of surgery until the date of death. All statistical analyses and data management were performed by an independent statistician using R- software (Rx64 version 4.0.3)^12,13^. The population were divided into EVH vs OVH and data was coded for analysis to blind the statistician. To investigate the effect of the two surgical techniques (OVH vs EVH) on mortality, we used Inverse Probability of Treatment Weights (IPTW) to balance demographics and preoperative comorbidities between the two groups.

Missing variables were imputed with multiple imputation using all baseline variables in Table 1, plus intervention (open/endoscopic), duration in the study (days), year of operation and survival status in February 2020 (dead/alive). Ten multiply imputed data were created. To assess the intervention effect, each imputed data was weighted using IPTW, intervention effect was calculated in each weighted dataset using cox proportional hazard regression in which time to event was regressed against intervention. Results were pooled using Rubin’s rule.

#### IPTW model

The propensity scores^14^ were estimated using a logistic regression model in which intervention assignment (OVH or EVH) was regressed on the 25 covariates and their pairwise interactions, as listed in Table 1. Also restricted cubic splines with 5 knots were entered for age, BMI and logistic EuroSCORE. Pairwise interactions between multi-level (more than 2 levels) categorical variables were removed as they had very low balance at some levels and created large weights. Stabilised weights to assess Average Treatment Effect (ATE) were calculated for each imputed set.

#### Balance diagnostics in weighted datasets

It has been suggested that standardised differences in excess of 10% may be indicative of meaningful imbalance in a covariate between the two groups and less than 10% is negligible imbalance^15^. Balancing criteria were considered as standardised mean difference less than 10%, variance ratio between treatment groups for continuous variables less than 2 and Kolmogorov–Smirnov thresh-old of less than 4% for equality of the continuous variables between treatment groups. The balance was assessed for each variable in Table 1, squared and cubic order of continuous variables and 2x2 interaction between continuous variables^16^.

## Results

### Pre-operative data

During the study period, a total of 13,794 patients underwent EVH and 13,230 underwent OVH for CABG surgery. Patient characteristics and pre-operative data are included in Table 1. The majority of patients in the EVH group underwent surgery at Harefield hospital, although the ratio of OVH cases was more evenly split between hospitals. Table 1is the summary of baseline and pre-operative comorbidities. Add EuroSCORE had a skewed distribution and therefore logistic (Add EuroSCORE) was used. Continuous variables are reported by mean(SD) and categorical variables with count (percentage). A total of 7 out of 25 preoperative variables had standardised difference above 10%, indicating imbalance between the two groups (Table 1). IPTW was applied to 10 imputed datasets. Mean stabilized weights for the 10 imputed data were 1.006-1.007 with standard deviations 0.66-0.67. Stabilized weights were all smaller than 10. Maximum absolute standardized differences between the two intervention groups in weighted datasets was 0.036 (Table 1), maximum KS difference for continuous variables was 0.017 and maximum variance ratio for continuous variables was 1.04.

### Peri-operative data

Peri-operative details are displayed in Table 2. The length of hospital stay is similar for both groups at median 6 days (p=0.86). Significant differences are observed in the type of surgery undertaken, with a greater number of EVH patients also receiving valve surgery or additional other cardiac surgery compared to the OVH group (p<0.001). A greater number of EVH patients also had the mammary artery used compared to the OVH group (p=0.009).

### Mortality

**Figure 2:**
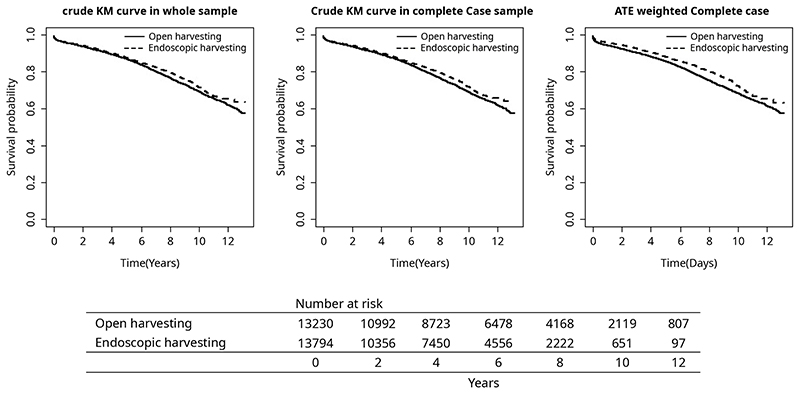
Kaplan-Meier survival curves for the study cohort.

The primary outcome measure for the study was post-operative mortality. Across the follow-up period of the study (median 1656 days for EVH and 2191 days for OVH), deaths from any cause occurred for 13.8% of EVH patients and 20.8% of OVH patients. In-hospital death occurred at similar rates (2.3% EVH vs. 2.1% OVH, p=0.12; Table 3). The crude hazard ratio of death (EVH to OVH, HR (95% CI)) was computed as 0.902 (0.850, 0.957) for raw sample data, 0.892 (0.839, 0.948) for complete case data and 0.851 (0.789, 0.919) for weighted complete case data. Kaplan-Meier plots for the raw data, complete case data and weight complete case data are depicted in Figure 2, with greater survival in the EVH group compared to the OVH group (p<0.001 for all).

Since the missing data was not completely at random, multiple imputation was used to impute missing data for Euroscore and previous MI. Each imputed set was weighted and hazard ratio for mortality was obtained for each weighted dataset. Results were pooled using Rubin’s rule.

Overall hazard ratio (95% CI) of death EVH:OVH was 0.823 (0.767, 0.884). As a sensitivity analyses for unmeasured confounders, once those with weights outside overlapped region and once those with weights above 99 percentile and below 1 percentile of all weights were excluded, remaining data were re-weighted and hazard ratios were calculated and pooled again. Hazard ratio (95% CI) from excluding non-overlapped weights was 0.829 (0.772, 0.889). Hazard ratio (95% CI) from excluding lower and upper 1% weights was 0.837 (0.778, 0.900). There seem to be a slight bias for unmeasured confounder, but nevertheless the difference between the two interventions stays significant.

### Secondary outcome measures

Post-operative complications and requirement for repeat operation is displayed in Table 3. Repeat operation was required more frequently in the EVH group (3.9% vs. 2.7%, p<0.001), however this was not as a result of graft occlusion (EVH vs. OVH: 0.1% vs. 0.2%). There was a significant difference in post-operative arrhythmias between the groups (p<0.001). Whilst a similar proportion of patients were free of arrhythmias in each group (EVH vs. OVH: 79.6% vs. 79.5%, respectively), there was a difference in the nature of arrhythmias recorded between the groups, with a greater requirement for permanent pacing in the EVH group (1.4% vs. 0.9%). A similar effect was observed in post-operative gastrointestinal complications with 98.2% free from problems in both groups. However, the nature of the complications observed differed between the groups (p=0.003). Finally, pulmonary complications were observed at a higher rate in the EVH group compared to OVH (14.7% vs. 12.8%, p<0.001), driven predominantly by a more common occurrence of collapse and consolidation (5.8% vs. 2.9%). Importantly, there were also a significantly greater number of surgical site infections in the OVH group, driven primarily by leg wound infection (p<0.001).

## Discussion

Endoscopic conduit harvesting has become the preferred surgical technique for the harvesting long saphenous vein and radial artery harvesting in many cardiac surgery centres. A meta-analysis of 43 randomised controlled trials and observational studies of 27,789 patients supported the superiority of the EVH technique for multiple endpoints including wound infection, cosmetic healing, patient satisfaction, myocardial infarction and mortality^17^.

Primarily, our study indicates that EVH is non-inferior to OVH over a mean follow-up time of 1656 and 2191 days for EVH and OVH respectively, with hazard of all-cause mortality significantly lower in the EVH group. Dacey and colleagues reported for 4 years mortality outcome (n=8,542) that the use of EVH was associated with a significantly reduced risk of mortality (HR 0.74; 95% CI 0.60 to 0.92; p=0.007) after adjusting for relevant covariates, and this finding endured even after propensity score analysis^18^. Furthermore, a recent publication from Zenati and colleagues reported no significant difference in their study randomised control trial between EVH and OVH mortality (12.0% vs. 13.2% respectively), at 4.7 years median follow up (HR: 0.90; 95% CI, 0.65-1.25; p=0.52)^19^. The PREVENT-IV trial reported that EVH patients had increased mortality compared to OVH group (HR 1.52; 95% CI 1.13 to 2.04; p=0.01)^20^, but that was not the case in our study or those previously referenced^3,5,19,21^. Sadly, we were unable to collect the exact cause of death for our cohort of patients due to the geographical catchment area and patients who moved out of the area.

As part of the secondary analysis in our study, the surgical site wound infection rates were compared between the groups. Two hospitals have an established wound clinic, and all patients are followed up until 6 to 8 weeks. Our results demonstrated that EVH patients have fewer leg wound infections than those in the OVH group (0.4% vs 2.6%). Similar results regarding leg wound infections have been reported in the literature indicating that EVH is far superior to OVH^6,9,11,18,19,22,23^. Only symptomatic patients who have returned to the source hospital were included for the analysis of repeat graft surgery data analysis (n=8 patients in the EVH group and n=21 in the OVH group). These findings align strongly with the recent publication by Zenati and colleagues, who also demonstrate that there is no significant difference in rate of repeat re-vascularisation post CABG surgery between the EVH and OVH groups (HR, 0.79; 95% CI, 0.54 – 1.17; p=0.25)^19^. Current evidence supports the use of endoscopic vein harvesting in multi vessel coronary artery disease^6,9,24^ and it is based on the effectiveness of these techniques, that the International Society of Minimally Invasive Cardiac Surgery consensus statements now recommend (class 1, Level B) that endoscopic vein harvesting should be standard of care for patients^25^.

There are many concerns regarding EVH, and the initial ones are related to the practitioner experience and their ability. There are further concerns regarding the creation of a carbon dioxide tunnel around the harvest conduit^26–28^ and our previous study has demonstrated that there was no impact by the tunnel^29^. The final concerns are regarding the more likely use of thigh vein compared to the calf vein and its possible impact on long-term graft patency, but it is not proved yet. A few studies have suggested a reduction in vein graft patency on angiographic follow up^20,30^ and it causes concerns among many surgeons. We hope our study and others will reassure surgeons and referring cardiologists that the use of EVH does not impact on hard outcome measures from patient standpoint. We do not have angiographic data to allay fears on that point of graft patency, but this could only be answered by a large, randomised control trials which is unlikely to be funded in this financial climate and patients are most interested in survival and lack of need for reintervention and less interested in angiogram findings. Our study captures a real-world experience from three large volume centres over a long-time frame and we hope this helps with a gradual acceptance with this approach in the UK and Europe.

A single centre experience with 10 years outcomes has been published^21^ but this is the first multicentre case series with a survival follow-up until 10 years. We do believe the transition from OVH to EVH should be managed in each institution carefully and would support the development of training standards as there is increasing evidence that practitioner experience and techniques do affect outcomes^6–8,19^ and unless these are standardised there will be likely to be a variation in outcomes.

### Study limitations

This is an observational retrospective study rather than a prospective randomised controlled trial. Propensity score matching requires that the sample of untreated subjects be larger than the sample of treated subjects. Ideally, there should be substantially more untreated subjects than treated subjects. Therefore, for this study we have used IPTW, which considered weights based on the propensity score to create a synthetic sample in which the distribution of measured baseline covariates is independent of treatment assignment. A subject’s weight is equal to the inverse of the probability of receiving the treatment that the subject received. Propensity score weighting assumes that there is no unmeasured confounding factor (e.g. all confounding factors have been considered in the model).

Another important limitation is that there are not angiographic details for the entire cohort of patients. Limited angiographic data was available only for symptomatic patients who have come back to the base hospital for reintervention, and these are listed on the Table 4. Patients who have attended peripheral hospitals were not captured due to large geographical catchment areas. It is difficult to accurately capture major adverse cardiac events in both groups due to lack of routine planned follow up in specialist cardiac centres. Importantly, we need to take into consideration that it is a multicentre observational study where there is likely to be a degree of variations in vein harvesting and surgical techniques by different operators.

## Conclusion

Our study has concluded that the use of endoscopic vein harvesting does not appear to adversely affect survival out to 10 years compared to the open vein harvesting and in this non-randomised group the long-term survival appears to be better with EVH. As the early results related to wound infection and cosmesis favour EVH, we do believe that this approach should be adopted more widely in the interest of patients.

## Figures and Tables

**Table 1:** Demographics and pre-operative variables.

	EVH (n=13794)	OVH (n=13230)	Absolute standardised differences (original data)	Maximum Absolute standardised differences (weighted imputed data)
**Hospital, n (%)**			0.726	0.029
*Blackpool*	*1920* (13.9)	4027 (30.4)		
*Harefield*	9808 (71.1)	4909 (37.1)		
*Wythenshawe*	2066 (15.0)	4294 (32.5)		
**Age, mean (SD)**			0.006	0.009
***EVH+OVH:* 66.73 (10.09)**	66.76 (10.17)	66.70 (10.02)		
**BMI, mean (SD)**			0.014	0.009
***EVH+OVH:* 28.29 (4.85)**	28.32 (4.91)	28.26 (4.80)		
**Log (Add Euro Score), mean (SD)**			0.049	0.012
***EVH+OVH:* 1.39 (0.76) n=1629 missing**	1.41 (0.73) n=985 missing	1.37 (0.78) n=644 missing		
**Sex, n (%)**			0.014	0.005
*Male*	11122 (80.6)	10595 (80.1)		
*Female*	2672 (19.4)	2635 (19.9)		
**Urgency, n (%)**			0.034	0.009
*Elective*	8866 (64.3)	8378 (63.3)		
*Urgent or Salvage*	218 (1.6)	264 (2)		
*Emergency*	4710 (34.1)	4588 (34.7)		
**Smoking, n (%)**			0.090	0.004
*Never*	5553 (40.3)	4757 (36)		
*Ex-smoker*	6881 (49.9)	7143 (54)		
*Current smoker*	1360 (9.9)	1330 (10.1)		
**Diabetic, n (%)**			0.138	0.016
*No*	9326 (67.6)	9669 (73.1)		
*Diet therapy*	457 (3.3)	501 (3.8)		
*Oral therapy*	2918 (21.2)	2168 (16.4)		
*Insulin therapy*	1093 (7.9)	892 (6.7)		
**Hypertension**	10320 (74.8)	7896 (59.7)	0.327	0.033
**Ventilated preoperatively**	25 (0.2)	59 (0.4)	0.047	
**CCS, n (%)**			0.144	0.011
*No angina*	2040 (14.8)	1846 (14)		
*No limitation of physical activity*	951 (6.9)	1356 (10.2)		
*Slight limitation of physical activity*	5377 (39)	5400 (40.8)		
*Marked limitation of ordinary physical activity*	3909 (28.3)	3448 (26.1)		
*Symptoms at rest or minimal activity*	1517 (11)	1180 (8.9)		
**NYHA, n (%)**			0.128	0.014
*No limitation of physical activity*	*3302* (23.9)	3902 (29.5)		
*Slight limitation of physical activity*	6582 (47.7)	5775 (43.7)		
*Marked limitation of physical activity*	3359 (24.4)	3101 (23.4)		
*Symptoms at rest or minimal activity*	551 (4)	452 (3.4)		
**Previous MI, n (%)**			0.043	0.011
*No MI*	8348 (60.5)	8184 (61.9)		
*One episode of MI*	4708 (34.1)	4320 (32.7)		
*Two or more episode*	632 (4.6)	693 (5.2)		
**Previous PCI, n (%)**			0.096	0.010
*No previous PCI*	11314 (82)	11023 (83.3)		
*PCI>24 hours before surgery*	2208 (16)	1833 (13.9)		
*PCI<24 hours before surgery*	80 (0.6)	176 (1.3)		
*PCI > 24 hours before surgery*	192 (1.4)	198 (1.5)		
**Renal disease, n (%)**			0.696	0.023
*Normal*	9455 (68.5)	12456 (94.1)		
*Moderate*	3471 (25.2)	606 (4.6)		
*Severe*	868 (6.3)	168 (1.3)		
**Pulmonary disease, n (%)**			0.110	0.012
*No chronic pulmonary disease*	12054 (87.4)	11538 (87.2)		
*Asthma*	89 (0.6)	169 (1.3)		
*COPD/emphysema*	88 (0.6)	193 (1.5)		
*Chronic pulmonary disease require treatment*	1563 (11.3)	1330 (10.1)		
**Neurological past medical history, n (%)**			0.012	0.004
*No history of neurological disease*	12819 (92.9)	12257 (92.6)		
*TIA*	525 (3.8)	517 (3.9)		
*CVA*	450 (3.3)	456 (3.4)		
**Peripheral vascular disease, n (%)**	1475 (10.7)	1562 (11.8)	0.035	0.002
**Extent of Coronary Artery Disease Pre surgery, n (%)**			0.079	0.036
*No vessel >50% diameter stenosis*	161 (1.2)	130 (1)		
*One vessel with >50% diameter stenosis*	1666 (12.1)	1385 (10.5)		
*Two vessels with >50% diameter stenosis*	3343 (24.2)	2960 (22.4)		
*Three vessels with > 50% diameter stenosis*	8624 (62.5)	8755 (66.2)		
**LVEF, n (%)**			0.059	0.013
*Good LVEF > 50%*	10644 (77.2)	9884 (74.7)		
*Moderate LVEF30-49%*	2656 (19.3)	2789 (21.1)		
*PoorLVEF<30%*	494 (3.6)	557 (4.2)		
**Inotropes given preoperatively, n (%)**	75 (0.5)	122 (0.9)	0.044	0.009
**Left Main Stem disease, n (%)**	3380 (24.5)	3408 (25.8)	0.029	0.004
**Pre surgery Nitrates administration, n (%)**			0.144	0.008
*No*	13228 (95.9)	12696 (96)		
*Until day of operation*	535 (3.9)	355 (2.7)		
*Within one week of operation*	31 (0.2)	179 (1.4)		
**Preoperative cardiogenic shock, n (%)**	60 (0.4)	90 (0.7)	0.033	0.003
**Use of preoperative IABP, n (%)**	283 (2.1)	213 (1.6)	0.033	0.003

**Table 2:** Perioperative data.

	EVH (n=13,794)	OVH (n=13,230)	p-value
**Cardiopulmonary bypass time, mean (SD)**	71.60 (65.56) n=5 missing	76.43 (57.13) n=7 missing	<0.001^
**Aortic cross clamp time, mean (SD)**	47.40 (43.73) n=9 missing	47.99 (40.26) n=14 missing	0.49^
**Type of surgery, n (%)**			
*CABG*	11118 (80.6)	11341 (85.7)	<0.001*
*CABG+valve*	2000 14.5)	1605 (12.1)	
*CABG + valve + other*	365 (2.6)	137 (1.0)	
*CABG + Other*	311 (2.3)	147 (1.1)	
**Mammary artery used, n (%)**	12863 (93.3)	12229 (92.4)	0.00983
**Heart valves, n (%)**			
*Not used*	11460 (83.1)	11490 (86.8)	<0.001*
*One valve replaced*	2151 (15.6)	1629 (12.3)	
*Two valves replaced*	183 (1.3)	111 (0.8)	
**Cardiopulmonary bypass used, n (%)**	9379 (68.0)	10296 (77.8)	<0.001*

* Chi^2^ test.

^ Mann-Whitney U test. Continuous variables are median (IQ) [range]. EVH: Endoscopic Vein Harvesting; OVH: Open Vein Harvesting

**Table 3:** Secondary outcomes.

	EVH	OVH	p-value*
**Length of stay (days), mean (SD)**	9.19 (9.81)	9.09 (9.70)	0.86^
**Hospital death, n (%)**	324 (2.3)	273 (2.1)	0.120
**Surgical site infection, n (%)**			
*None*	13281 (96.3)	12557 (94.9)	<0.001*
*Superficial sternal wound infection*	266 (1.9)	194 (1.5)	
*Leg wound infection*	55 (0.4)	341 (2.6)	
*Not specified*	45 (0.3)	38 (0.3)	
*Superficial sternal and leg wound infection*	0 (0.0)	2 (0.0)	
*Deep sternal and leg wound infection*	5 (0.0)	23 (0.2)	
*Deep sternal wound infection*	106 (0.8)	50 (0.4)	
*Radial artery site harvest infection*	1 (0.0)	2 (0.0)	
*Mediastinal wound infection*	35 (0.3)	23 (0.2)	
**Repeat operation, n (%)**			
*Not necessary*	13260 (96.1)	12879 (97.3)	<0.001*
*Reoperation for bleeding or cardiac tamponade*	383 (2.8)	260 (2)	
*Sternum re-suturing*	28 (0.2)	15 (0.1)	
*Surgery for deep sternum wound infection*	36 (0.3)	16 (0.1)	
*Re-operation for other and cardiac valve problems*	78 (0.6)	38 (0.3)	
*Redo surgery for graft occlusion*	8 (0.1)	21 (0.2)	
*Sternum re-suturing and Surgery for deep sternum wound infection*	0 (0.0)	1 (0.0)	
*Surgery for deep sternum wound infection and Re-operation for other and cardiac valve problems*	1 (0.0)	0 (0.0)	
**Post arrhythmias, n (%)**			
*No*	10975 (79.6)	10524 (79.5)	<0.001
*Atrial fibrillation/flutter required intervention*	2435 (17.7)	2393 (18.1)	
*Supraventricular tachycardia required intervention*	82 (0.6)	88 (0.7)	
*Ventricular Fibrillation/Tachycardia required intervention*	109 (0.8)	111 (0.8)	
*Permanent Pacing required*	193 (1.4)	114 (0.9)	
**Post Pulmonary complications, n (%)**			
*No*	11769 (85.3)	11541 (87.2)	<0.001
*Not specified*	301 (2.2)	461 (3.5)	
*Full tracheostomy*	118 (0.9)	113 (0.9)	
*Reintubation & ventilation*	259 (1.9)	189 (1.4)	
*Pulmonary embolism*	33 (0.2)	8 (0.1)	
*Chest infection*	492 (3.6)	525 (4)	
*Collapse & consolidation*	805 (5.8)	379 (2.9)	
*Acute Respiratory Distress Syndrome*	17 (0.1)	14 (0.1)	
**Post Gastrointestinal complications, n (%)**			
*No*	13551 (98.2)	12996 (98.2)	0.00337
*Other not specified*	125 (0.9)	160 (1.2)	
*Pancreatitis*	24 (0.2)	16 (0.1)	
*Peptic ulceration*	10 (0.1)	10 (0.1)	
*Bleeding but not specified*	55 (0.4)	25 (0.2)	
*Ischaemic bowel, laparotomy*	29 (0.2)	23 (0.2)	

* Chi^2^ test and

^ Mann-Whitney U test. EVH: Endoscopic Vein Harvesting; OVH: Open Vein Harvesting

**Table 4:** Number of angiograms, graft occlusions and percutaneous coronary interventions. Data available for Wythenshawe and Blackpool symptomatic patients who had follow up.

	OVH, n (%) (N=8321)	EVH, n (%) (N=3986)	P*
Angiogram	203/8321 (2.4)	160/3986 (4)	<0.001
Percutaneous coronary Intervention	121/203 (59.6)	93/160 (58.1)	0.972
Vein graft blockage	87/203 (42.9)	70/160 (43.8)	1
LIMA blocked	38/203 (18.7)	37/160 (23.1)	0.399
Medical therapy	184/203 (90.6)	154/160 (96.3)	0.177

EVH: Endoscopic Vein Harvesting; OVH: Open Vein Harvesting

## Data Availability

Underlying data The raw patient data were obtained from the Royal Brompton and Harefield, Wythenshawe and Blackpool hospitals. The hospitals and Edgehill University gave permission to conduct analysis on the data for this study only and not future studies. For further research using these datasets, additional permission must be sought from these hospitals. Researchers who require access to the raw data will need to contact the audit/ research department of each hospital; contact details are available as follows: Royal Brompton and Harefield Research office, https://www.rbht.nhs.uk/research/research-office, Wythenshawe, mft.rd@manchester.ac.uk; Blackpool, https://www.bfwh.nhs.uk/onehr/research-development/. Intermediary data can be found in the article.

## Plain language summary

Use of keyhole vein removal technique has been increasing for coronary artery bypass surgery for last two decades. However, some surgeons remain worried about the quality and long-term effect of the vein tube removed using keyhole method compared to traditional Open Vein Harvesting (OVH). The aim of this study was to perform a retrospective analysis of the outcomes between keyhole and OVH from three UK centres with 10 years follow-up. In total, 27,024 patients underwent coronary artery bypass surgery with long saphenous vein harvested by either keyhole (n=13,794) or OVH (n=13,230) over a period of 2007–2019. The median follow-up time was 4.54 years for keyhole method and 6.00 years for OVH. Death from any cause occurred in 13.8% of the keyhole group versus 20.8% in the OVH group over the follow-up period. In conclusion, the keyhole surgery survival is not as bad as we hypothesised.
