# Supplementary material for: A multicentre review comparing long term outcomes of endoscopic vein harvesting versus open vein harvesting for coronary artery bypass surgery
Source: NIHR Open Res. 2021 Jul 8;1:11. [Version 2] doi: 10.3310/nihropenres.13215.1 (PMC7612280; doi:10.3310/nihropenres.13215.1)
Supplement: Peer review history

# Reviewer report: Reviewer response for version 1

**DOI:** 10.3310/nihropenres.14331.r28424

**Published:** 2021-11-15

**Authors:** Sridhar Rathinam

**Affiliations:** 1 Department of Thoracic Surgery, Glenfield Hospital, University Hospitals of Leicester Trust, Leicester, UK

Thank you for an interesting article looking at an important aspect of cardiac surgical care. Congratulations to the authors for investigating the LSV harvesting by EVH and OVH in the United Kingdom.

 May I seek a few clarifications as well as suggest some revisions:

1. The authors declare registration in Edgehill and Wythensawe, was this study not registered in the other two hospitals whilst sharing patient data even anonymized into a pooled analysis. This needs clarification.
2. For a study of this significant impact, it would be ideal to have the interventions done in the peripheral hospitals as this will offer a true re intervention data.
3. Please use hospitals names consistently.
4. Can the authors clarify why there was less IMA use in OLV group was it higher risk or elderly patients as this may have an impact on the results?
5. The authors state challenges in declaring cause of death, surely this data is available with the GPs?
6. Please briefly describe or reference the difference in the anatomical sites of harvest between OLV and EVH which is described in discussion but not in the methods.
7. Can the authors describe the percentage of harvest done by trainee surgeons and trainee SCPs as the experience impacts on outcomes?
8.  Have the authors done a subgroup analysis of patients seen in base hospital to confirm the conclusion (i.e full angiographic and symptom data)?
9. Please consider naming the  the institution eg Blackpool Victoria Hospital. 
10. Do the authors mean National census rather than censor?
11. Surgical technique: Suggest change to "patients who had their veins retrieved by...".
12. Discussion: Suggest change "We do not have angiographic data to allay fears on that point of graft patency, but this could only be answered by a large, randomised control trials which is **challenging in this **financial climate."
13. Consider rephrasing the following "...patients are most interested in survival and lack of need for reintervention and less interested in angiogram findings." - the presence and lack of symptoms and reintervention are highlighted by the angiogram findings so this is a bold statement.

I hope these queries and suggestions are useful. Thank you for the opportunity.

Is the work clearly and accurately presented and does it cite the current literature?

Partly

If applicable, is the statistical analysis and its interpretation appropriate?

Yes

Are all the source data underlying the results available to ensure full reproducibility?

No source data required

Is the study design appropriate and is the work technically sound?

Yes

Are the conclusions drawn adequately supported by the results?

Partly

Are sufficient details of methods and analysis provided to allow replication by others?

Yes

Reviewer Expertise:

Cardiothoracic trials. Lung cancer, Device and intervention RCTs, LVRS

I confirm that I have read this submission and believe that I have an appropriate level of expertise to confirm that it is of an acceptable scientific standard, however I have significant reservations, as outlined above.

# Reviewer report: Reviewer response for version 1

**DOI:** 10.3310/nihropenres.14331.r28407

**Published:** 2021-10-11

**Authors:** Joel Dunning

**Affiliations:** 1 Department of Cardiothoracic Surgery, The James Cook University Hospital, Middlesbrough, UK

This is an excellent and very large study by a well respected group of surgeons from 3 leading hospitals in the UK. There is very long term follow up and this in my view is the absolute best study that could be performed without randomisation or angiography. This does very much support the safety of EVH and provides potential evidence for superiority for this technique without any additional risk to the patient. 

 There are some important systematic biases between the groups and in the selection of patients for EVH or OVH but the authors have addressed this and this would be the case in any cohort study such as this. 

 I think that this should provide the basis for an NIHR application for a Randomised Study in this area as this is certainly the best that could possibly be done as a study with the non-randomised data currently available.

Is the work clearly and accurately presented and does it cite the current literature?

Yes

If applicable, is the statistical analysis and its interpretation appropriate?

Yes

Are all the source data underlying the results available to ensure full reproducibility?

Yes

Is the study design appropriate and is the work technically sound?

Yes

Are the conclusions drawn adequately supported by the results?

Yes

Are sufficient details of methods and analysis provided to allow replication by others?

Yes

Reviewer Expertise:

Cardiothoracic Surgery

I confirm that I have read this submission and believe that I have an appropriate level of expertise to confirm that it is of an acceptable scientific standard.

# Reviewer report: Reviewer response for version 1

**DOI:** 10.3310/nihropenres.14331.r28409

**Published:** 2021-09-27

**Authors:** Anson Cheung

**Affiliations:** 1 Centre for Heart Valve Innovation, St. Paul’s Hospital, University of British Columbia, Vancouver, BC, Canada

Congratulations to the authors for the interesting study which investigate the benefits of minimally invasive SVG harvesting over open harvesting in patients undergoing CABG.

 I have few minor revisions:

1. Tables can be improved, data could be better reported limiting to the most important ones, making the table more self-explanatory.
2. Authors correctly state that operator's expertise can make a difference in terms of long term results since the manipulation of the graft might affect its patency. However the authors do not investigate this important aspect. I believe operators' experience should be included in the analysis.
3. The reported death rate is not cardiovascular death, it is not clear if patients died in relation to the surgery or for not surgery related causes. Since one of the main issues related to EVH is possible excessive manipulation of the graft that could cause anticipated graft occlusion authors should report CV death also, specifically MI deaths and HF deaths.

Is the work clearly and accurately presented and does it cite the current literature?

Partly

If applicable, is the statistical analysis and its interpretation appropriate?

Yes

Are all the source data underlying the results available to ensure full reproducibility?

No source data required

Is the study design appropriate and is the work technically sound?

Yes

Are the conclusions drawn adequately supported by the results?

Partly

Are sufficient details of methods and analysis provided to allow replication by others?

Yes

Reviewer Expertise:

Off-pump CABG with minimally invasive SVG and radial artery harvesting, LVAD, heart transplant, TAVR

I confirm that I have read this submission and believe that I have an appropriate level of expertise to confirm that it is of an acceptable scientific standard, however I have significant reservations, as outlined above.
